# Effects of the Lipid Profile, Type 2 Diabetes and Medication on the Metabolic Syndrome—Associated Gut Microbiome

**DOI:** 10.3390/ijms23147509

**Published:** 2022-07-06

**Authors:** Gratiela Gradisteanu Pircalabioru, Janie Liaw, Ozan Gundogdu, Nicolae Corcionivoschi, Iuliana Ilie, Luciana Oprea, Madalina Musat, Mariana-Carmen Chifiriuc

**Affiliations:** 1 Research Institute of University of Bucharest (ICUB), 300645 Bucharest, Romania; carmen.chifiriuc@bio.unibuc.ro; 2 Faculty of Infectious & Tropical Diseases, London School of Hygiene and Tropical Medicine, Keppel Street, London WC1E 7HT, UK; janie.liaw@lshtm.ac.uk; 3 Bacteriology Branch, Veterinary Sciences Division, Agri-Food and Biosciences Institute, Belfast BT9 5PX, UK; nicolae.corcionivoschi@afbini.gov.uk; 4 Faculty of Bioengineering of Animal Resources, Banat University of Agricultural Sciences and Veterinary Medicine—King Michael I of Romania, 300645 Timisoara, Romania; 5 Gral Medical Clinic, 031424 Bucharest, Romania; ilieiu@yahoo.com; 6 National Institute of Endocrinology C.I. Parhon, 011863 Bucharest, Romania; lucianaoprea92@yahoo.com (L.O.); mdmusat@yahoo.com (M.M.); 7 Department of Endocrinology, Carol Davila University of Medicine and Pharmacy, 020021 Bucharest, Romania; 8 Romanian Academy, 010071 Bucharest, Romania

**Keywords:** metabolic syndrome, diabetes, microbiome, dysbiosis, metformin

## Abstract

Metabolic syndrome (MetSyn) is a major health problem affecting approximately 25% of the worldwide population. Since the gut microbiota is highly connected to the host metabolism, several recent studies have emerged to characterize the role of the microbiome in MetSyn development and progression. To this end, our study aimed to identify the microbiome patterns which distinguish MetSyn from type 2 diabetes mellitus (T2DM). We performed 16S rRNA amplicon sequencing on a cohort of 70 individuals among which 40 were MetSyn patients. The microbiome of MetSyn patients was characterised by reduced diversity, loss of butyrate producers (*Subdoligranulum*, *Butyricicoccus*, *Faecalibacterium prausnitzii*) and enrichment in the relative abundance of fungal populations. We also show a link between the gut microbiome and lipid metabolism in MetSyn. Specifically, low-density lipoproteins (LDL) and high-density lipoproteins (HDL) display a positive effect on gut microbial diversity. When interrogating the signature of gut microbiota in a subgroup of patients harbouring both MetSyn and T2DM conditions, we observed a significant increase in taxa such as *Bacteroides*, *Clostridiales*, and *Erysipelotrichaceae*. This preliminary study shows for the first time that T2DM brings unique signatures of gut microbiota in MetSyn patients. We also highlight the impact of metformin treatment on the gut microbiota. Metformin administration was linked to changes in *Prevotellaceae*, *Rickenellaceae*, and *Clostridiales*. Further research focusing on the microbiome-metabolome patterns is needed to clarify the exact association of various gut microbial communities with the progression of T2DM and the occurrence of various complications in MetSyn patients.

## 1. Introduction

The widespread changes in lifestyle and dietary patterns brought by Westernisation have led to an increased prevalence of metabolic syndrome (MetSyn) [1]. Being the result of a cluster of risk factors such as obesity, dyslipidemia, hyperglycemia, hyperuricemia, and hypertension, MetSyn often progresses to increased cardiovascular risk and ailments such as non-alcoholic fatty liver (NAFLD) and type 2 diabetes mellitus (T2DM) [2]. MetSyn is the result of a wide array of triggers including chronic inflammation, insulin resistance, and oxidative stress, all of them being also associated with gut dysbiosis [3,4]. Thus, microbiota changes induced by unhealthy diets activate inflammatory cascades [5]. The microbiota of obese people was suggested to harvest more energy from the diet, a fact which leads to higher body mass index and insulin resistance [6].

In this complicated equation, recent studies have emerged to attribute a potential role for the gut microbiome in MetSyn. A recent multi-ethnic population study reported that MetSyn patients were enriched in *Enterobacteriaceae* and low in *Turicibacter* and *Peptostreptococcaceae* [7]. Qin et al. (2021) reported that microbiome changes in MetSyn patients (decreased abundance of *Alistipes onderdonkii*, *Clostridium asparagiforme*, *C. citroniae*, *C. scindens*, *Roseburia intestinalis*, and *Bacteroides thetaiotaomicron*) may increase inflammation and play a part in MetSyn pathogenesis by inhibiting short-chain fatty acids (SCFAs) production [8]. Perhaps the most compelling evidence regarding the role of the microbiome in MetSyn comes from faecal microbiota transplant (FMT) studies. Indeed, the transfer of microbiota from lean donors to obese individuals with MetSyn resulted in an increased abundance of butyrate-producing microbes as well as an increase in insulin sensitivity within six weeks [9].

Here, we profile the gut microbiome composition and diversity in Metsyn patients via high-throughput sequencing of the V3–V4 region of 16S ribosomal DNA (rDNA) coupled with Real-Time PCR. We report changes in taxa associated with SCFAs production as well as disturbances in the mycobiota associated with MetSyn. Finally, we perform a comparative analysis of the intestinal microbiome in MetSyn patients with and without T2DM as well as between patients receiving different types of medication (statins and metformin). 

## 2. Results

### 2.1. Clinical Characteristics of the Enrolled Subjects

We enrolled a total of 56 women and 14 men in this study and among them, 40 subjects were diagnosed with MetSyn. The healthy control group harboured 30 healthy individuals who had fewer than two MetSyn-associated markers. The two groups were similar in terms of age or gender composition. Body mass index (BMI), glycated haemoglobin (HbAc), and tryglyceride levels (TG) were significantly higher in the case of MetSyn patients compared to the control group (Table 1).

### 2.2. Diversity Patterns in MetSyn Patients

**Figure 1:**
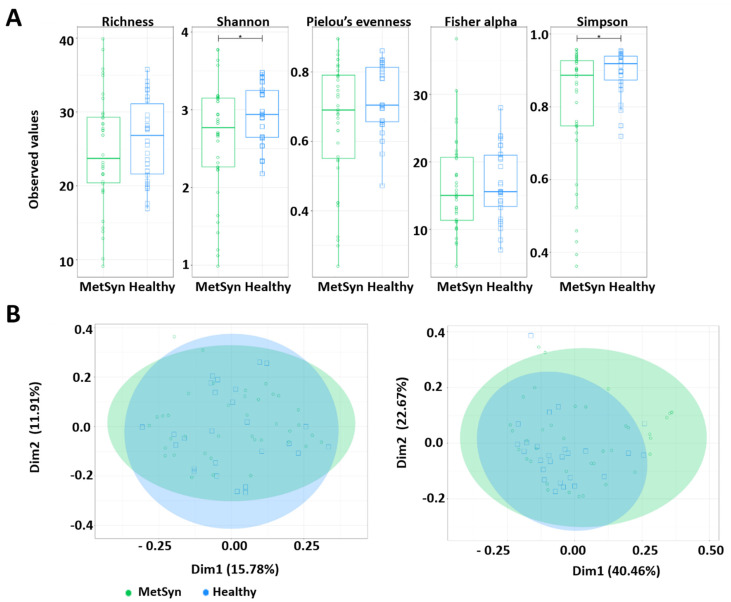
Microbial diversity and community structure for MetSyn vs. healthy controls. (**A**) Alpha diversity measurements between MetSyn patients and healthy controls; (**B**) Beta diversity using *Bray-Curtis* (left) and weighed *UniFrac* (right).

The faecal microbiota of the 70 subjects enrolled in the study (40 patients with MetSyn and 30 control individuals) was evaluated by Illumina MiSeq amplicon sequencing. First, several alpha diversity indices were calculated to analyse the microbiome differences in the MetSyn patient samples compared to the healthy controls. *Richness*, *Shannon*, *Pielou’s evenness*, *Fisher alpha*, and *Simpson* were calculated for all samples to establish the alpha diversity patterns in healthy individuals and patients with MetSyn (Figure 1A). No statistically significant differences were found when using *Richness*, *Shannon*, *Pielou’s* *evenness*, and *Fisher alpha* to assess the microbial richness between healthy and MetSyn individuals. *Simpson* diversity which considers the number of species, as well as their relative abundance, was lower in the case of MetSyn patients (*p* < 0.05). This is in line with previous reports whereby patients with metabolic disorders harbour decreased microbial diversity [8]. 

The β diversity differences between bacterial communities found in MetSyn patients were calculated using the *Bray-Curtis* metric (which considers the species abundance count) and Weighted *UniFrac* metric (the phylogenetic distance between the branch lengths of OTUs observed in different individuals based on the abundances of OTUs) and visualised by principal coordinate analysis (Figure 1B). No statistical differences were observed between healthy controls and MetSyn patients. 

To further analyse the data, we performed LCBD which shows how significantly different microbial population structures are from the average (with LCBD values differing from the mean LCBD values representing outliers). LCBD analysis was performed by using: the *Hellinger* distance (abundances) (Supplementary Figure S1A); unweighted *UniFrac* (phylogenetic distance) (Supplementary Figure S1B); and weighted *UniFrac* (phylogenetic distance weighted by abundance) dissimilarities (Supplementary Figure S1C). No differences were observed between MetSyn and control patients for *Hellinger* distance and unweighted *UniFrac*. However, when considering weighted *UniFrac* (phylogeny and abundance), the data shows the healthy control data being furthest away from the average. 

To assess the factors triggering microbial community diversity, the values of NRT (Nearest Relative Index) and NTI (Nearest Taxon Index) were measured. While positive NRI/NTI values show phylogenetic clustering due to the environment, reduced NRI/NTI is indicative of phylogenetic overdispersion, with limited input from environmental filtering (Supplementary Figure S1D). No statistical differences were observed between healthy controls and MetSyn patients. In all samples tested, the core microbiome consisted of genera *Faecalibacterium* and *Bacteroides* (Supplementary Figure S2A). Differences in terms of types of genera were also presented in Supplementary Figure S2B. 

In parallel, to test if there was any difference in microbiome community structure between MetSyn and healthy controls, PERMANOVA was performed (Table 2). Here, with *Bray-Curtis* distance, 29.9% of the variability in community structure is explained by HDL levels. Although no statistical significance was detected when using *UniFrac* distance, when using weighted *UniFrac*, 38.7% value of the variability in community structure was explained by the HDL levels (Table S1). 

Subset analysis was performed to identify a subset of OTUs that describes approximately the similar beta diversity between samples as all the OTUs (Table 3). Here, we have obtained a reduced feature set (OTUs) in the sample space that is deriving the change. Only one group comparison is displayed (MetSyn versus healthy control), whereas the remaining results are shown within the Supplementary files. Additionally, after imploding to the subset of genera, PERMANOVA analysis was carried out to verify if this subset still has discriminatory power (in terms of grouping). Here, we identified *Clostridiales*, *Bacteroides*, *Ruminococcaceae*, *Christensenellaceae*, *Bifidobacterium*, *Lachnospiraceae*, and *Proteobacteria* as the subset of OTUs which differentiate healthy controls vs. MetSyn patients.

**Figure 2:**
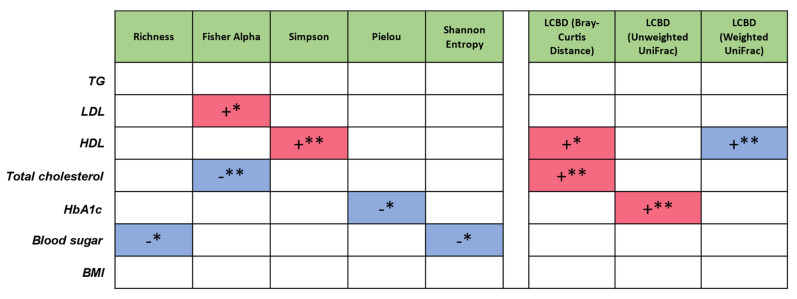
Subset regression where red and blue represent the significant positive and negative beta coefficients that were consistently selected in different regression models. As an example, Blood sugar is having a negative influence on increasing microbial diversity (for 2/5 diversity metrics). Likewise, Total cholesterol is having a positive influence on LCBD (*Bray-Curtis*), shifting the microbial community structure away from the average; *, *p* < 0.05; **, *p* < 0.01.

To measure the influence of extrinsic parameters on microbial community structure, whereas PERMANOVA analysis displays the degree of impact on the microbiome population structure in terms of variability, to achieve directionality as to whether an increase or decrease in these parameters can lead to variation in the properties of microbiome metrics, subset regressions on one-dimensionality realisation of the microbiome (alpha diversity—*Richness*, *Simpson*, *Pielou’s evenness* index, *Shannon*, *Fisher alpha*; LCBD beta diversity—*Bray-Curtis*, unweighted *UniFrac*, weighted *UniFrac*) were performed. Subset regression against different sources of variation (“TG”, “LDL”, “HDL”, “Total cholesterol”, “HbA1c”, “Blood sugar”, “BMI”) was carried out by testing all the combination of all these variations and then selecting the best model according to statistical criteria (adjusted R^2^, etc.) (Figure 2). Red and blue signify whether the predictors harbour a positive or a negative influence, respectively within the regression model. LDL and HDL display a positive effect on microbial diversity. Total cholesterol, HbA1c, and blood sugar display a negative effect on microbial diversity. Total cholesterol (*Bray-Curtis*) and HbA1c (*UniFrac*) displayed a positive impact on the microbial community structure away from the average. HDL also had a positive impact when considering microbial numbers (*Bray-Curtis*), but a negative one when considered in conjunction with the type of OTUs (weighted *UniFrac*) (Figure 2). 

Genera and families identified at varying levels of abundance for healthy controls and MetSyn groups include *Faecalibacterium*, *Bacteroides*, *Prevotella* 9, *Ruminococcaceae*, Eubacterium, *Clostridium*, *Alistipes*, Succinivibrio, Anaerotruncus, Barnesiella, Sutterella, *Subdoligranulum* and *Akkermansia* (Supplementary Figure S2B). Genera that were differentially expressed between healthy control and MetSyn patients were identified (Supplementary Figure S2C). Taxa differential analysis showed that three OTUs were distinct between healthy controls and MetSyn patients (Supplementary Figure S2C). MetSyn patients were enriched in *Ruminococcaceae* UCG-005 (adjusted *p*-value = 0.0054285) and *Clostridiales* (adjusted *p*-value = 0.033447) and lower in *Bacteroidaceae DJF_B220* (adjusted *p*-value = 0.00038163).

**Figure 3:**
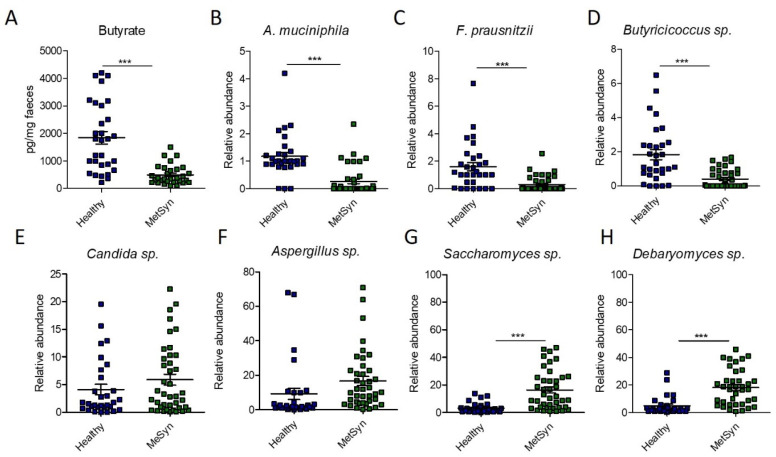
Microbiome changes in MetSyn patients (*n* = 40) versus healthy controls (*n* = 30). (**A**) Butyrate quantification in faecal samples; The relative abundance of *A. muciniphila* (**B**), *F. praunsitzii* (**C**), *Butiricicoccus* spp. (**D**), *Candida* spp. (**E**), *Aspergillu* spp. (**F**), *Saccharomyces* sp. (**G**), and *Debaryomyces* spp. (**H**) in faecal samples collected from healthy individuals and MetSyn patients; *** *p* < 0.0001, Mann–Whitney test.

Since *Clostridiales* are important producers of SCFAs, especially butyrate [10], we next investigated the levels of faecal butyrate in our patient cohort. However, we found that MetSyn patients had significantly decreased levels of butyrate (Figure 3A). The difference observed in the case of butyrate levels prompted us to further investigate the relative abundance of other microbes producing butyrate in the gut. Compared to the healthy controls, MetSyn patients were also low in *Subdoligranulum* (Supplementary Figure S2B), another member of the microbiome which produces butyrate [11]. In addition, we analysed via qRT-PCR the abundance of other taxa important for gut health, metabolism, and SCFA production such as *Akkermansia muciniphila*, *Faecalibacterium prausnitzii*, and *Butyricicoccus* spp. Importantly, we found that MetSyn patients were significantly depleted in all three of the aforementioned taxa (Figure 3B–D). The Gram-negative bacterium *Akkermansia muciniphila* has been described as a beneficial taxon in the gut, protecting against metabolic diseases and colitis, even though it is not butyrate, but an acetate and propionate producer [12]. Our data also shows that the microbiota of individuals with MetSyn is significantly lower in *A. muciniphila*. Butyrate not only fuels the cells in the gut lining and exhibits anti-inflammatory traits, but it also harbours antifungal properties [13]. Hence, we hypothesised that the imbalance in SCFAs identified in MetSyn subjects may trigger gut dysbiosis and subsequent changes in the gut mycobiome. Therefore, we investigated the relative abundance of several fungal taxa including *Candida* spp., *Aspergillus* spp., *Saccharomyces* spp., and *Debaryomyces* spp. using qRT-PCR. Generally, MetSyn was linked with a higher abundance for all four analysed fungal taxa. *Candida* spp. had a relatively high abundance in the case of MetSyn subjects but this was not statistically significant (Figure 3E). Particularly, only *Saccharomyces* and *Debaryomyces* were significantly increased in patients with metabolic disorders (Figure 3G,H). In addition, *Aspergillus* spp. tended to be in higher abundance in faecal samples of MetSyn patients but this difference was not statistically significant (Figure 3F).

**Figure 4:**
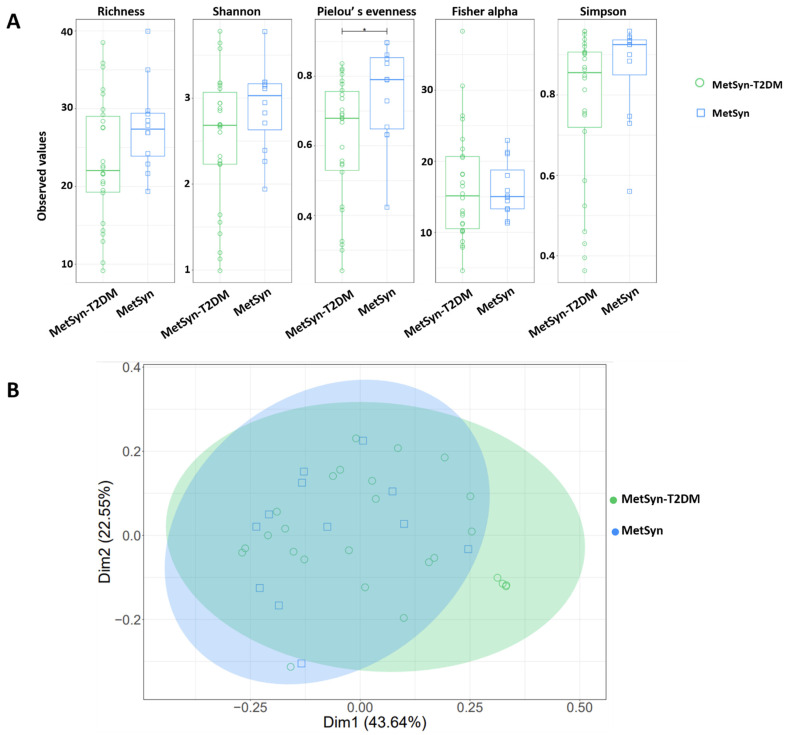
Microbial diversity and community structure for MetSyn and MetSyn-T2DM patients. (**A**) alpha diversity measurements; (**B**) Beta diversity analysis-weighed *UniFrac*.

**Figure 5:**
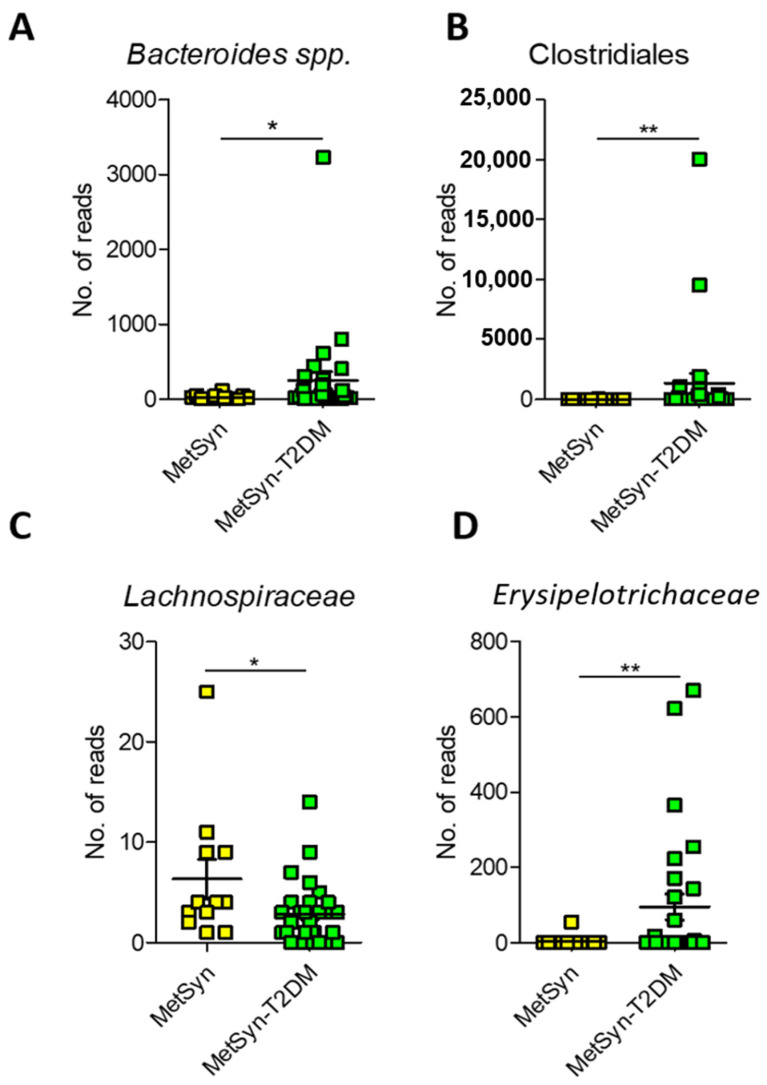
Microbiome signatures in T2DM compared to MetSyn. A. Number of reads for *Bacteroides* (**A**), *Clostridiales* (**B**), *Lachnospiraceae* (**C**), and *Erysipelotrichaceae* (**D**). *, *p* < 0.05; **, *p* < 0.01.

Since MetSyn is a cluster of conditions that have been linked to various ailments (i.e., T2DM and heart disease) we next aimed to see whether there are any specific microbiome signatures associated with diabetes. MetSyn patients who also had diabetes exhibited a significant increase in HbAc (** *p*-value = 0.0016) and in blood glucose levels (*** *p* < 0.0001) (Supplementary Table S2)., MetSyn patients with T2DM (MetSyn-T2DM) exhibited a decrease in microbiome alpha diversity (Figure 4A) as revealed by *Pielou’s evenness* metric. No significant changes were observed in terms of beta diversity regardless of the matrices used (Figure 4B and Supplementary Figure S3) or for subset analysis (Table S3). However, when interrogating the abundance of different OTUs we observed that T2DM was associated with significant changes in taxa such as *Bacteroides*, *Clostridiales*, *Lachnospiraceae*, and *Erysipelotrichaceae* (Figure 5A–D). We also checked whether there was a difference in butyrate levels (Supplementary Figure S4A), *A. muciniphila* (Supplementary Figure S4B), *F. prausnitzii* (Supplementary Figure S4C), and *Butyricicoccus* spp. (Supplementary Figure S4D) in case of MetSYN-T2DM patients (Supplementary Figure S4A–D). However, we did not find significant differences when comparing the levels of butyrate or the aforementioned taxa. We next set to investigate the changes associated with a fungal population such as *Candida* spp., *Aspergillus* spp., *Saccharomyces* spp., and *Debaryomyces* spp. (Supplementary Figure S4E–H). Among the tested fungi, only *Candida* spp., was found to be significantly increased in the MetSyn group (*p*-value = 0.0008) (Supplementary Figure S4E).

Next, we aimed to elucidate the impact of medication taken by the analysed patients on the gut microbiome. For instance, statins can significantly decrease the risk of cardiovascular disease in MetSyn patients by inducing alterations in lipid levels and possibly by decreasing inflammation. In our cohort, 26 patients were taking statins at the time of sample analysis. No significant differences were found in terms of microbial diversity, subset analysis of OTUs and differential taxa analysis for the patients taking statins compared to the treatment naive MetSyn patients (Supplementary Figure S5, Supplementary Table S4). However, metformin treatment did impact the structure of the microbiome in MetSyn patients. We observed changes in terms of alpha diversity measured by *Fisher alpha* and *Richness* index in the case of MetSyn patients taking metformin (Figure S6A) but not in case of *Shannon*, *Pielou’s evenness*, or *Simpson* metrics (Supplementary Figure S6). OTU differential analysis showed that metformin treatment was positively associated with taxa such as *Rikenellaceae* RC9 gut group (padj = 0.004221) and negatively correlated with *Prevotella* 9 (padj = 0.00422), *Bacteroides* (padj = 0.004724), *Prevotellaceae* (padj = 0.005852) and *Clostridiales* (padj = 0.011694) (Table 4). Beta diversity analysis of the microbiome measured by *Bray-Curtis*, *UniFrac*, and weighted *UniFrac* (Supplementary Figure S6) and subset analysis revealed no significant changes in the case of metformin treatment (Table S5).

## 3. Discussion

Even though the definition of a healthy microbiome is still missing, diverse medical conditions have been linked to certain microbiota patterns [14]. An altered microbiota generally characterised by loss of diversity and an enrichment of opportunistic pathogens has been reported for obesity, T2DM, and MetSyn [15]. In the case of MetSyn, detailed analyses of the microbiome variance depending on the type of comorbidities, and treatment regimens are still needed to improve patient outcomes. Complementary to existing pharmaceutical options, interventions targeting the patient microbiota may ameliorate disease or decrease the morbidity associated with MetSyn. Indeed, the use of probiotics has been reported to modulate the microbiome and to subsequently enhance insulin sensitivity, hence improving metabolic health [6]. The diversity of gut bacteria regarding microbial numbers and their comparative abundance evenness is a potent indicator of host health [16,17]. Hence, lower alpha diversity (also known as intra-individual diversity) is an indicator of dysbiosis. Importantly, dysbiosis has been linked to several features of MetSyn [18]. 

Within this study, we show a link between the gut microbiome and lipid metabolism in MetSyn. Specifically, LDL and HDL display a positive effect on gut microbial diversity. Interestingly, when considering LCBD, HDL levels have a positive impact when only considering microbial numbers (*Bray-Curtis*), but a negative one when considered in conjunction with the type of OTUs (weighted *UniFrac*). Importantly, low HDL values are associated with an increased risk of cardiovascular disease [19]. Other studies have also shown a link between HDL and the gut microbiota [20,21,22]. Hence, manipulation of gut the microbiota may serve as an ideal therapeutic approach for improving HDL function as well as cardiovascular risk.

Based on metagenomics and qPCR data, we highlight here several aspects related to the gut microbiome in MetSyn, particularly in MetSyn coupled with T2DM. To sum up, we show here that MetSyn patients have lower microbial diversity when compared to healthy controls and that in terms of taxa abundance, they are low in the gut beneficial microorganisms such as *F. praunsnitzii*, *A. muciniphila*, and *Subdoligranulum*. *Subdoligranulum* is a strictly anaerobic, non-spore-forming Gram-negative microorganism which produces the SCFA butyrate, a metabolite with multiple health benefits [10,23]. Several diseases, including T2DM or inflammatory bowel diseases, were associated with decreased abundance of butyrate producers like *Subdoligranulum* [24]. Importantly, *Subdoligranulum* has been shown to be positively correlated with HDL cholesterol and negatively correlated with glycated haemoglobin (HbA1c) [10].

MetSyn patients were characterised by a decrease in three markers of gut health—butyrate levels and abundance of *F. prausnitzii* and *A. muciniphila. F. prausnitzii* strains are considered major butyrate producers in the human intestine [25] and require acetate for the production of butyrate through the butyryl CoA: acetate CoA-transferase pathway [26]. Importantly, the mucin degrading *A. muciniphila* bacterium increases the acetate and propionate pool stimulating the syntrophic growth of *F. prausnitzii*, subsequently stimulating butyrate production [27,28]. MetSyn subjects were found to be low in butyrate, *F. prausnitzii*, and *A. muciniphila* and these findings are in accordance with other published studies [8,29,30,31,32,33,34]. Indeed, metagenomics studies performed on in independent human cohorts consistently revealed a decrease in butyrate-producing bacteria in individuals with metabolic diseases [8,34]. Hence, restoration of butyrate-producing bacteria and butyrate levels might provide new treatment options for MetSyn and T2DM. Indeed, a low abundance of *A. muciniphila* has been correlated with obesity, hypertension, and treatment naive T2DM [35,36]. Moreover, a recent randomised crossover clinical study (double-blind, placebo-controlled) using daily oral *A. muciniphila* supplementation resulted in significantly improved insulinemia, insulin sensitivity, and plasma total cholesterol [37]. 

We show here that MetSyn is associated with a low abundance of these beneficial taxa and increased levels of *Debaryomyces*, a fungus recently reported to inhabit inflamed intestinal tissue and can lead to impaired mucosal healing [38]. Moreover, we report here that three OTUs distinguish the microbiome of MetSyn patients from that of healthy individuals and these are *Clostridiales*, *Ruminococcaceae* UCG-005, and *Bacteroidaceae DJF_B220*.

The increase of *Clostridiales* in MetSyn and T2DM was also reported by other research groups. Indeed, compared with nondiabetic individuals, patients with T2DM have an increased percentage of *Clostridiales* spp., *Lactobacillus* spp., *Streptococcus mutans*, and Betaproteobacteria class and a decreased proportion of *F. prausnitzii* and *Roseburia intestinalis* [39]. An increased presence of *Ruminococcaceae* UCG 005 was reported in acute coronary syndrome compared to the healthy control group, as well as increased serum trimethylamine N-oxide (TMAO) concentration [40]. It was suggested that *Ruminococcaceae* UCG 005 may be predictive biomarkers for cardiovascular events development [40]. Nevertheless, the results of a recent study by Tomizawa et al. (2021) showed that *Ruminococcaceae* UCG 005 may also be affected by age, gender, and genetics, and therefore further research concerning these factors needs to be considered [41]. 

*Bacteroidaceae* were reported to be increased in high-fat diet-fed mice [42] and decreased in obese individuals [43]. Here, we report that MetSyn is associated with an enrichment in the abundance of the *Bacteroidaceae* bacterium DJF_B220, a taxon which remains poorly understood in terms of its relation to human disease. So far, an increased abundance of *Bacteroidaceae DJF_B220* was reported in patients with nephrolithiasis [44].

Findings from our study indicate that the MetSyn microbiome differs from that of healthy people, while the addition of the T2DM condition brings additional signatures. Hence, patients with MetSyn and T2DM have a gut microbiota enriched in *Bacteroides*, *Clostridiales*, *Erysipelotrichaceae*, and low in *Lachnospiraceae*. *Lachnospiraceae* belong to and are part of the gut microbiota core being among the main SCFAs producers [45]. Although members of this bacterial family have been shown to generate beneficial metabolites for the host, their abundance was also reported to be increased in various diseases. A metagenome-wide association study of gut microbiota in T2DM patients carried out by Qin et al. (2012) reported a high abundance of *Lachnospiraceae* in diabetics [46]. Nevertheless, this study was performed on a Chinese cohort and it is well-known that geographic location and diet hold an impact on microbiome composition. Specifically, *Blautia* (a member of the *Lachnospiraceae* family) has gained attention due to its contribution to alleviating inflammatory and metabolic diseases [47,48]. Nevertheless, the intestinal abundance of *Blautia* species is influenced by geography, age, genotype, diet, and other diseases [49,50,51].

Blooming of the *Erysipelotrichaceae* family has been reported enriched in ileal Crohn’s disease [52], colorectal cancer [53] as well as in diet-induced obese animals [54] and in obese individuals [55]. We report here a significant enrichment of *Erysipelotrichaceae* and *Bacteroides* in the case of MetSyn patients with associated T2DM. MetSyn-T2DM patients from the cohort described were enriched in *Bacteroides* abundance. However, the role of *Bacteroides* in diabetes is a controversial one. While several studies reported that *Bacteroides* is inversely proportional to diabetes risk [56,57,58,59], other studies found a positive correlation for different species [60,61,62]. In diabetic mice, administration of *Bacteroides acidifaciens* and *B. uniformis* prevented obesity and improved insulin sensitivity suggesting that *Bacteroides* may have beneficial effects [63,64]. These conflicting findings may be caused by the use of different animal models and the analysis of the microbiome at various stages of the disease.

Due to its efficacy, safety, tolerability, and low cost, metformin is the most frequently administered medication to treat patients with T2DM [65] and it was recently reported to affect the gut microbiome. An example of metformin’s effects on the gut microbiota is the increased abundance of the mucin-degrading *A. muciniphila*, a protective ally against the development of metabolic diseases [66]. Moreover, metformin intake was associated with an increase in SCFAs production [67]. Metformin administration was reported to increase *E. coli* levels and decrease *Intestinibacter bartlettii* [68,69]. However, in our study, we did not find any correlation between metformin intake and *Enterobacteriaceae* and *Intestinibacter* abundance probably due to the small sample size and the demographic characteristics of the cohort analysed. In healthy mice, metformin targets the gut microbiome by increasing the abundances of *Verrucomicrobiaceae*, *Akkermansia* spp., *Clostridium* spp., *Ruminococcaceae*, *Alistipes* spp., and *Rikenellaceae* [70].

In the work presented here, we show that metformin is associated with an increase in the *Rickenellaceae* RC9 gut group. *Rikenellaceae* RC9 gut group, belonging to the *Rikenellaceae* family, plays an important role in the digestion of crude fiber and can produce propionate, acetate, and/or succinate as fermentation end-products [71]. In rats, this taxonomic group was shown to be associated with a beneficial phenotype after treatment with polyphenols to alleviate doxorubicin cardiotoxicity [72]. Members of the *Rikenellaceae* RC9 gut group were reported to protect against oxidative stress, thereby lowering inflammation. [73]. Nevertheless, in a rat model of isoproterenol induced ischemia, *Rickenellaceae* RC9 group was correlated to a higher risk of acute myocardial ischemia and it was suggested to be involved in lipid metabolism [74]. These contradictory findings suggest that the *Rickenellaceae* RC9 gut group still needs to be analysed in terms of its connection to human health and the gut barrier function and permeability.

So far, the role of members of the *Prevotellaceae* family within the gut microbiota and their effects on the host is not fully understood. While some studies report *Prevotella* as beneficial microbes enriched in people consuming a vegetarian diet [75,76], others have linked them with insulin resistance, diabetes, and gut inflammation [77,78]. *Prevotellaceae* were shown to be increased in the gut microbiota of T2DM patients [79] and recently, Díaz-Perdigones et al. [80] (2022) reported that metformin treatment lowers the abundance of this taxon [80]. In our cohort, metformin treatment led to a significant reduction of *Prevotellaceae* and *Prevotella* 9.

This initial exploratory research performed on a cohort of 70 individuals points out the existence of dysbiosis in MetSyn patients and of specific signatures in MetSyn patients with T2DM. One limitation of the study was the relatively small sample used but we plan to extend our research to bigger cohorts. Importantly, we excluded from our analysis patients who had a history of COVID-19, considering that SARS-CoV-2 infection significantly alters the gut microbiome [81]. Given the heterogeneity in the overall microbial composition observed in the participants, further recruitment would enable the detection of additional correlations between gut microbiota and MetSyn and T2DM pathogenesis.

For the first time, this preliminary study reports the existence of specific microbiota compositional alterations in MetSyn patients with associated T2DM compared to individuals with MetSyn alone. More investigations focusing on the microbiome-metabolome patterns are needed to clarify the exact association of various gut microbial communities with the progression of T2DM.

## 4. Materials and Methods

### 4.1. Patients

A total of 70 subjects were enrolled in this study. Among them, 40 were MetSyn patients from the National Institute of Endocrinology “C.I. Parhon”, Bucharest, Romania, and 30 were healthy volunteers. All participants received and signed an informed consent, and the Ethical Committee approved the study (CEC reg. no 235/9.10.2019).

Inclusion criteria for participating in the study were: (1) diagnosis of MetSyn using the International Federation Of Diabetes criteria 2006 [82], respectively waist > 94 cm (men)/>80 cm (women) along with (2) the presence of two or more of the following: blood glucose greater than 100 mg/dL or diagnosed diabetes, blood triglycerides > 150 mg/dL or drug treatment for elevated triglycerides, HDL cholesterol < 40 mg/dL in men/<50 mg/dL in women or drug treatment for low HDL-C, blood pressure > 130/85 mmHg or drug treatment for hypertension, ages 25 to 75 years.

The exclusion criteria were: coexistence of other chronic inflammatory and systemic autoimmune diseases; pregnancy, antibiotic treatment in the past month, steroid therapy in the past 3 months; neoplastic disease not in complete remission, history of COVID-19, and history of chronic infectious disease (i.e., tuberculosis, infections with HIV, HBV or HCV). Age, gender, and ethnicity matched healthy controls were enrolled based on the same exclusion criteria.

### 4.2. DNA Isolation

Stool samples were brought to the laboratory within 24 h after collection or, alternatively, were kept at −20 °C in a freezer until the study participants’ visit to the hospital. DNA was extracted from faecal samples using bead beating and the QIAamp DNA Stool Mini Kit (Qiagen, Germany). DNA concentration and purity were measured with a NanoDrop2000 and a Qubit 4 fluorometer (Thermo Fisher Scientific, Waltham, MA, USA).

### 4.3. Culture-Independent Analysis of Stool Samples

#### 4.3.1. 16S rRNA Amplification and Sequencing

16S metagenomic sequencing library generation was performed using Illumina guidelines (Illumina, San Diego, CA, USA). The 16S ribosomal primers V3 (TCGTCGGCAGCGTCAGATGTGTATAAGAGACAGCCTACGGGNGGCWGCAG) and V4 (GTCTCGTGGGCTCGGAGATGTGTATAAGAGACAGGACTACHVGGGTATCTAATCC) were used [83]. Microbiome sequencing was done using a v3 300 bp paired-end kit and the Illumina MiSeq platform.

#### 4.3.2. Bioinformatics

Paired-end reads were pre-processed following the guidelines from D’Amore et al. (2016) and Rognes et al. (2016) [83,84]. Sequencing reads were trimmed (average Phred quality score of 20 based on a sliding window approach) and filtered using Sickle v1.33 [85]. Error-correction on paired-end reads was done using BayesHammer from the Spades v3.1.1 assembler [86]. Forward and reverse reads were assembled into a single sequence spanning the V3–V4 16S rRNA region using PANDAseq (v2.11) [87].

Reads were further pooled, dereplicated, and arranged in order of declining abundance. Reads with only a single match were removed. In order to produce the abundance table by constructing OTUs, a representation of species, the VSEARCH v2.3.4 pipeline was used as described in http://github.com/torognes/vsearch/wiki/VSEARCH-pipeline) (accessed on 4 August 2021). Reads were clustered based on a 97% similarity. Cluster removal was performed by employing chimeric models generated from more abundant reads (the --uchime_denovo option in vsearch). The reference-based chimera filtering step (--uchime_ref option in vsearch) was carried out with the Silva gold database (https://www.mothur.org/w/images/f/f1/Silva.gold.bacteria.zip; accessed on 30 July 2021). The OTU table was generated after comparing the original barcoded reads to the cleaned OTUs. After generating a tab-delimited version of the OTU table (otus.fa; containing all of the OTU sequences) an otu_table.txt (the OTU abundance table) was constructed.

The resulting sequence file was uploaded into the QIIME2 pipeline [88]. Next, OTU taxonomy was assigned based on the SILVA SSU Ref NR database (v132) [89] and exported to a TSV format. Within QIIME2, a phylogenetic tree was created using FastTree (v2.1.10) and MAFFT (v7.310) [90,91]. The otu_table.txt and the taxonomy.tsv file were merged into a BIOM file within2 QIIME for subsequent analysis in R and phyloseq [92].

#### 4.3.3. Statistical Analysis of Sequencing Data

Statistical analyses were performed in R as previously described [93,94]. Output files from VSEARCH were analysed using the R vegan package for diversity measures [95]. Microbiome diversity within samples (alpha diversity) and between samples (beta diversity) was measured.

Alpha diversity was measured based on several indices: *Shannon* entropy, which measures the balance of a community within a sample (the higher the *Shannon* index, the more balanced the microbial community is); *Richness*—an estimation of species/features per rarefied sample; *Simpson* measures the community evenness from 0 to 1; *Pielou’s evenness* index- measures the evenness of a microbial community; *Fisher alpha* is an alternative diversity index. All of these aforementioned indices are the result of different analytical measurements and they depict various aspects of alpha diversity, whether emphasizing on predominant or on rare microbiome species.

For beta diversity analysis, three alternative distance matrices were used: (i) unweighted *UniFrac*, a phylogenetic distance metric (calculated using the phyloseq package) derived from the distances between samples by taking the fraction of the total of unshared branch lengths in the total of all branch lengths of the phylogenetic tree for the OTUs observed between samples (without taking into account their abundances); (ii) *Bray-Curtis* which is based on OTUs abundances as a dissimilarity measure; and (iii) weighted *UniFrac*, a phylogenetic distance metric that combined phylogenetic distance and relative abundances, highlighting dominant OTUs or taxa. 

In order to identify outliers in the beta diversity space, Local Contribution to Beta Diversity (LCBD) analysis [96] was done employing the LCBD.comp() from the adespatial package [97]. The unweighted *UniFrac* (phylogenetic distance), weighted *UniFrac* (phylogenetic distance weighted by abundance), and *Hellinger distance* (abundances) dissimilarities were used.

To distinguish if the structure of the microbial community was stochastic (overdispersion in the phylogenetic tree and triggered by competition), or deterministic (phylogenetic clustering and triggered by high environmental pressure), the distances in terms of phylogeny for every sample were determined via nearest taxa index (NTI) and net relatedness index (NRI). The NTI was calculated using mntd() and ses.mntd(), and the mean phylogenetic diversity (MPD) whereas NRI was calculated using mpd() and ses.mpd() functions from the picante package [98]. NTI and NRI are the negatives of the output from ses.mntd() and ses.mpd() and they measure the number of standard deviations separating the observed values from the mean of the null distribution.

The core microbiome was constructed using the R’s microbiome package and the recommendations given in from Shetty et al. (2017) [99] to find OTUs that are consistently prevalent in all samples with a reasonable abundance detection limit.

To obtain the minimal subset of species that can explain roughly the same beta diversity as compared to utilizing all of the OTUs in the sample space, we used the “BVSTEP” routine [100] to identify the highest correlation, in a Mantel test, by imploding the abundance table at genera level to an absolute minimal set of genera that maintain the beta diversity between samples. To run this algorithm, bvStep() (from the sinkr package) [101] was used as described in the author’s recent paper [93].

To allow the identification of genera that are significantly different between patient groups, DESeqDataSetFromMatrix() function from DESeq2 [102] package was utilised using the adjusted *p*-value significance cut-off of 0.05 and log2 fold change cut-off of 2. Bayesian shrinkage was further applied to acquire shrunken log fold changes subsequently employing the Wald test for acquiring adjusted *p*-values for multiple comparisons. DESeq2 identified changes on a local scale (in conjunction with beta diversity analysis) to highlight the genera responsible for the shift in microbial communities.

We wanted to get the directionality out by focusing on microbiome characteristics and the sources of variations that were identified for each sample. Subset regression of different microbiome metrics was performed against a set of explanatory variables (“TG”, “LDL”, “HDL”, “Total cholesterol”, “HbA1c”, “Blood sugar”, “BMI”), by selecting the best model (a subset of these variables) based on some statistical criteria (fit of regression, etc), with recommendations given in [103] and code available at http://www.sthda.com/english/articles/37-model-selection-essentials-in-r/155-best-subsets-regression-essentials-in-r/ (accessed on 3 June 2022). The R function regubsets() from leapspackage [104] was employed to point out different best models of different sizes, by specifying the option nvmax, set to the maximum number of predictors to incorporate the model. After acquiring the best possible subsets, the k-fold cross-validation comprised of dividing the data into k subsets. Each subset (10%) served successively as test data set and the remaining subset (90%) as training data. The average cross-validation error was then computed as the model prediction error. This was performed using a custom function based on the R’s train() function from the caret package [105]. Finally R’s tab_model() function from sjPlot package [106] was used to acquire the statistics for each model. In the majority of the figures containing boxplots, pair-wise ANOVA was done comparing two categories at a time. When significant differences were found (*p* ≤ 0.05), categories were joined together by a line and the significance levels were plotted on top of the plots (*: 0.01 ≤ *p* < 0.05; **: 0.05 ≤ *p* < 0.001; ***: *p* ≤ 0.001).

#### 4.3.4. qPCR

For qPCR, samples were diluted to an end concentration of 10 ng/μL. Primer sequences targeting the 16S rRNA gene [107] are presented in Table 5.

### 4.4. SCFAs Quantification

Sample preparation for metabolite analysis was performed as previously described [108]. Briefly, 0.2 g of feces were resuspended in 1 mL of phosphate saline buffer (pH 7.4) and further incubated at room temperature (2 min). After manual homogenization, samples were centrifuged (4000 rpm, 1 h, 4 °C) and the supernatant was further collected and centrifuged (6000 rpm, 30 min, 4 °C). Next, the supernatant was filtered using a minisart-GF filter membrane (Sartorius, Göttingen, Germany) and a Whatman-25mmGD/X0 filter (Millipore, Burlington, MA, USA). Butyrate levels were quantified using a commercial kit (Abbexa Ltd., Cambridge, UK) following the manufacturer’s instructions.

#### Statistical Analysis

Our study data are presented as mean ± SEM and were graphed using the GraphPad Prism 9.0 software. Sample size (n) denotes the biological replicates. The differences in microbial relative abundance were calculated using a non-parametric Mann-Whitney test. The * *p* < 0.05 was considered statistically significant. Statistical significance levels were *, *p* < 0.05; **, *p* < 0.01; ***, and *p* < 0.001.

## Figures and Tables

**Table 1:** Patient characteristics.

	Healthy Control (*n* = 30)	MetSyn (*n* = 40)	*p* Value
Gender	18 females, 12 males	34 females, 6 males	
Age	46 ± 13.98	52 ± 12.62	0.0645
BMI	24.7 ± 1.363448	32.4 ± 4.947618	*p* < 0.0001
HbAc (%)	5.4 ± 0.404021	6.6 ± 1.402163	*p* < 0.0001
TG mg/dL	89 ± 22.63105	124 ± 55.69321	0.0018
HDL mg/dL	64 ± 3.58	48.5 ± 8.290765	*p* < 0.0001
LDL mg/dL	98 ± 21.62	113.5 ± 36.78805	0.0438

**Table 2:** PERMANOVA analysis to investigate the influence of different parameters on microbial community structure. Here, using beta diversity (*Bray-Curtis*) distance metrics, 29.9% (R^2^ in the table given below) of the microbiome structure is explained. *, *p* < 0.05.

	Df	Sums of Sqs	MeanSqs	F.Model	R^2^	Pr (>F)
TG	1	0.2346	0.23461	0.68000	0.01065	0.854
LDL	1	0.3009	0.30090	0.87214	0.01366	0.626
HDL	1	0.6592	0.65924	1.91078	0.02994	0.012 *
Total cholest.	1	0.4702	0.47015	1.36272	0.02135	0.140
BMI	1	0.3451	0.34508	1.00019	0.01567	0.434
Residuals	58	20.0107	0.34501		0.90872	
Total	63	22.0207			1.00000	

**Table 3:** Subset analysis for MetSyn vs. control patients displaying subsets of OTUs along with the correlation of the beta diversity distances between these subsets and the full OTU table. The last column shows PERMANOVA statistics for these subsets pointing out their discriminatory power. R^2^ represents the percentage variability of these subsets in terms of groups.

Group Comparison	Subset No	Subset	Correlation of Subset with Full Table (R)	PERMANOVA Subsets (Groups)
MetSyn, Healthy	S1	*Clostridiales* + *Bacteroides* + *Ruminococcaceae* + *Christensenellaceae* + *Bifidobacterium* + *Lachnospiraceae* + *Proteobacteria*	0.00952	R^2^ = 0.822 (*p* > 0.05)
	S2	*Clostridiales* + *Bacteroides* + *Ruminococcaceae* + *Christensenellaceae* + *Bifidobacterium* + *Lachnospiraceae* + *Proteobacteria*	0.00965	R^2^ = 0.854 (*p* > 0.05)
	S3	*Clostridiales* + *Bacteroides* + *Ruminococcaceae* + *Christensenellaceae* + *Bifidobacterium* + *Lachnospiraceae* + *Proteobacteria*	0.00909	R^2^ = 0.874 (*p* > 0.05)

**Table 4:** Taxa differential of OTUs statistically modified when comparing Metformin treated MetSyn patients vs. naive patients. These are log 2-fold different and statistically significant.

OTU	baseMean	log2FoldChange	*p*-Value	padj	Upregulated
OTU_110 (*Rikenellaceae* RC9 gut group)	3.17082871	-2.35288	5.82 × 10^−5^	0.004222	Metformin
OTU_10 (*Prevotella 9*)	6.73972305	2.597327	3.64 × 10^−5^	0.004222	Control
OTU_43 (*Bacteroides*)	4.23676835	2.348046	0.0001	0.004725	Control
OTU_19 (*Bacteroides*)	3.34116511	−2.4488	0.00013	0.004725	Metformin
OTU_5 (*Prevotellaceae*)	6.38379068	2.389298	0.000202	0.005853	Control
OTU_11 (*Clostridiales*)	5.08918313	2.407934	0.000484	0.011694	Control

**Table 5:** Primer sequences targeting 16S rRNA gene.

Taxonomic Target	Sequence
*Butyricicoccus* spp.	ACCTGAAGAATAAGCTCC
	GATAACGCTTGCTCCCTACGT
*Akkermansia muciniphila*	GCG TAG GCT GTT TCG TAA GTC GTG TGT GAA AG
	GAG TGT TCC CGA TAT CTA CGC ATT TCA
rRNA16S	ACT CCT ACG GGA GGC AGC AGT
	ATT ACC GCG GCT GCT GGC
*F. prausnitzii*	CCCTTCAGTGCCGCAGT
	GTCGCAGGATGTCAAGAC
ARNr 18S	ATTGGAGGGCAAGTCTGGTG
	CCGATCCCTAGTCGGCATAG
*Saccharomyces* spp.	AGGAGTGCGGTTCTTTG
	TACTTACCGAGGCAAGCTACA
*Candida* spp.	TTTATCAACTTGTCACACCAGA
	ATCCCGCCTTACCACTACCG
*Debaryomyces* spp.	TAACGGGAACAATGGAGGGC
	CAACACCCGATCCCTAGTCG
*Aspergillus* spp.	GTGGAGTGATTTGTCTGCTTAATTG
	TCTAAGGGCATCACAGACCTGTT

## Data Availability

The data presented in this study are available on request from the corresponding author.

## Supplementary Materials

The following supporting information can be downloaded at: https://www.mdpi.com/article/10.3390/ijms23147509/s1.
