# Supplementary material for: Effects of the Lipid Profile, Type 2 Diabetes and Medication on the Metabolic Syndrome—Associated Gut Microbiome
Source: Int J Mol Sci. 2022 Jul 6;23(14):7509. doi: 10.3390/ijms23147509 (PMC9318871; doi:10.3390/ijms23147509)
Supplement: Supplementary file 1 [file ijms-23-07509-s001.zip › ijms-1716517-supplementary.pdf]

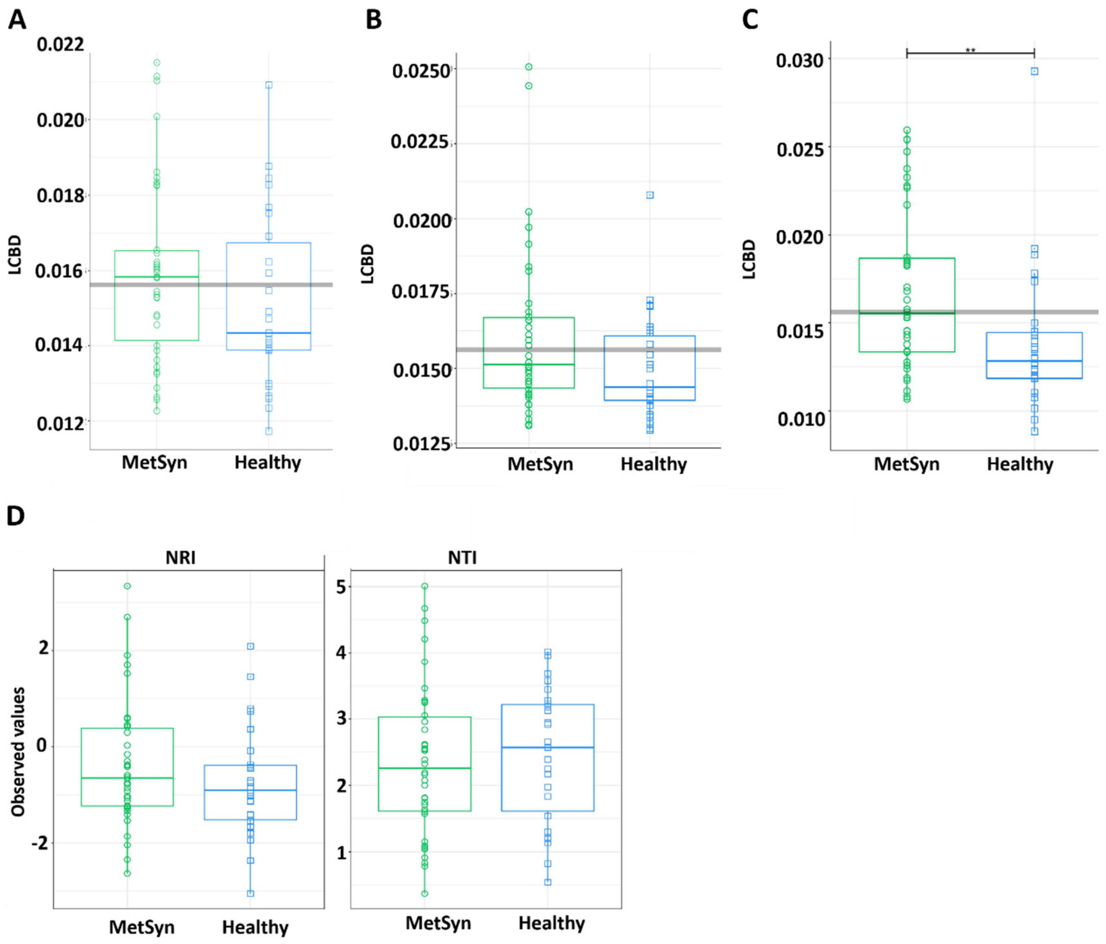

Figure S1. Microbiome structure in MetSyn patients versus healthy controls. A. LCBD in MetSyn patients and healthy controls; LCBD calculated by using sample wise proportional diversities: (A) *Hellinger* transform on the microbial counts; (B) unweighted *UniFrac* dissimilarity (phylogenetic distances only); and (C) weighted *UniFrac* dissimilarity (phylogenetic distances weighted with abundance counts), with all values summing up to 1. (D). NRI/NTI metrics; A relatively high value signifies a greater influence from environmental pressure on microbial population structure.

Table S1. PERMANOVA analysis to investigate the influence of different parameters on microbial community structure. Here, using beta diversity (weighted *UniFrac*) distance metrics, 38.7% (R^2 in the table given below) of the microbiome structure is explained.

	Df	SumsOfSqs	MeanSqs	F.Model	R^2	Pr(>F)
**TG**	1	0.1297	0.129748	1.90148	0.02896	0.102
**LDL**	1	0.0538	0.053775	0.78808	0.01200	0.544
**HDL**	1	0.1735	0.173541	2.54328	0.03874	**0.032 ***
**Total cholest.**	1	0.1141	0.114081	1.67188	0.02547	0.146
**BMI**	1	0.0508	0.050826	0.74486	0.01135	0.541
**Residuals**	58	3.9576	0.068235		0.88348	
**Total**	63	4.4796			1.00000	

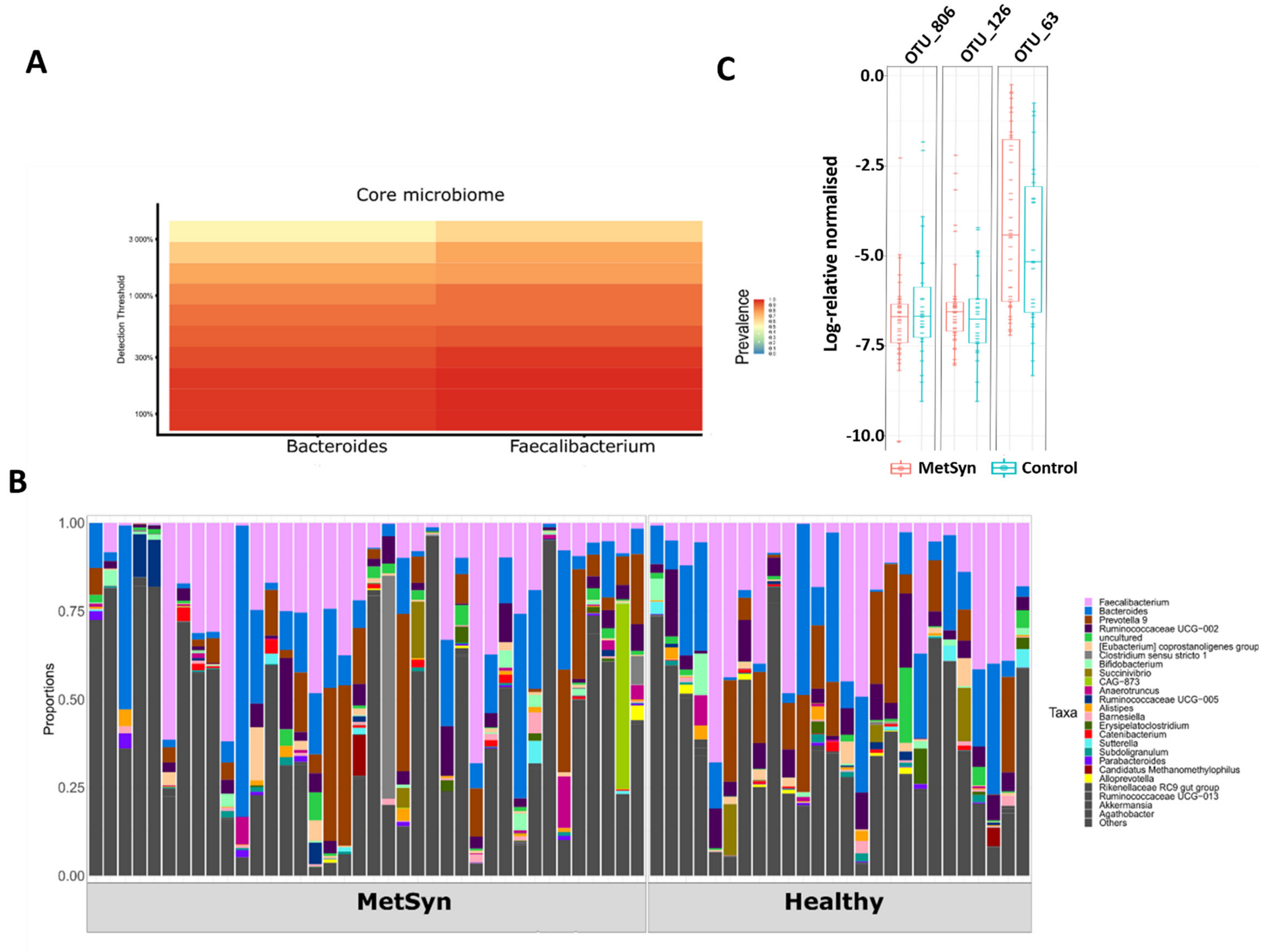

Figure S2. Microbiome analysis in MetSyn patients versus healthy controls. (A) The core microbiome in MetSyn patients and healthy controls; The figure shows at least 85% prevalent

OTUs ordered by lower abundance (left) to higher abundance (right). The y-axis (right) represents limit of detection in terms of relative abundance. (B) The top 25 most abundant genera representative of MetSyn and healthy controls. (C) Taxa differential of OTUs statistically modified when comparing MetSyn patients to healthy controls. Differential analysis of genera that are up/down-regulated between different groups (Adjusted P values ≤0.05) with at least log2 fold change from the base mean abundances for the samples considered in the first column; OTU_806 -*Bacteroidaceae* DJF_B220, OTU_126- *Ruminococcaceae* UCG-005; OTU_63- Clostridiales.

Table S2. Clinical parameters- MetSyn vs. MetSyn-T2DM

**Clinical parameter**	**MetSyn mean±SD**	**MetSyn-T2DM mean±SD**	**p-value**
**LDL**	114± 38.542	113.285± 28.063	0.9479
**HDL**	50.666±7.475	47.607±7.345	0.2372
**HbAc**	5.723± 0.567	7.106± 1.351	**** 0.0016**
**Blood glucose**	106± 12.315	136.571± 48.091	*****p<0.0001**
**Total Cholesterol**	191.833± 48.989	174.428± 46.299	0.2909
**TG**	130.333± 61.641	147.035± 50.846	0.3773

Table S3. **Subset analysis for MetSyn patients with and without diabetes displaying subsets of OTUs along with the correlation of the beta diversity distances between these subsets and the full OTU table**. The last column shows PERMANOVA statistics for these subsets pointing out their discriminatory power. R^2 represents the percentage variability of these subsets in terms of groups.

**Group Comparison**	**Subset No**	**Subset**	**Correlation of Subset with Full Table (R)**	**PERMANOVA Subsets (Groups)**
T2DM, no T2DM	S1	*Clostridiales* + *Bacteroides* + *Ruminococcaceae* + *Christensenellaceae* + *Bifidobacterium* + *Lachnospiraceae* + *Proteobacteria*	0.00952	R^2 = 0.848 ($p > 0.05$)
	S2	*Clostridiales* + *Bacteroides* + *Ruminococcaceae* + *Christensenellaceae* + *Bifidobacterium* + *Lachnospiraceae* + *Proteobacteria*	0.00965	R^2 = 0.824 ($p > 0.05$)
	S3	*Clostridiales* + *Bacteroides* + *Ruminococcaceae* + *Christensenellaceae* + *Bifidobacterium* + *Lachnospiraceae* + *Proteobacteria*	0.00909	R^2 = 0.847 ($p > 0.05$)

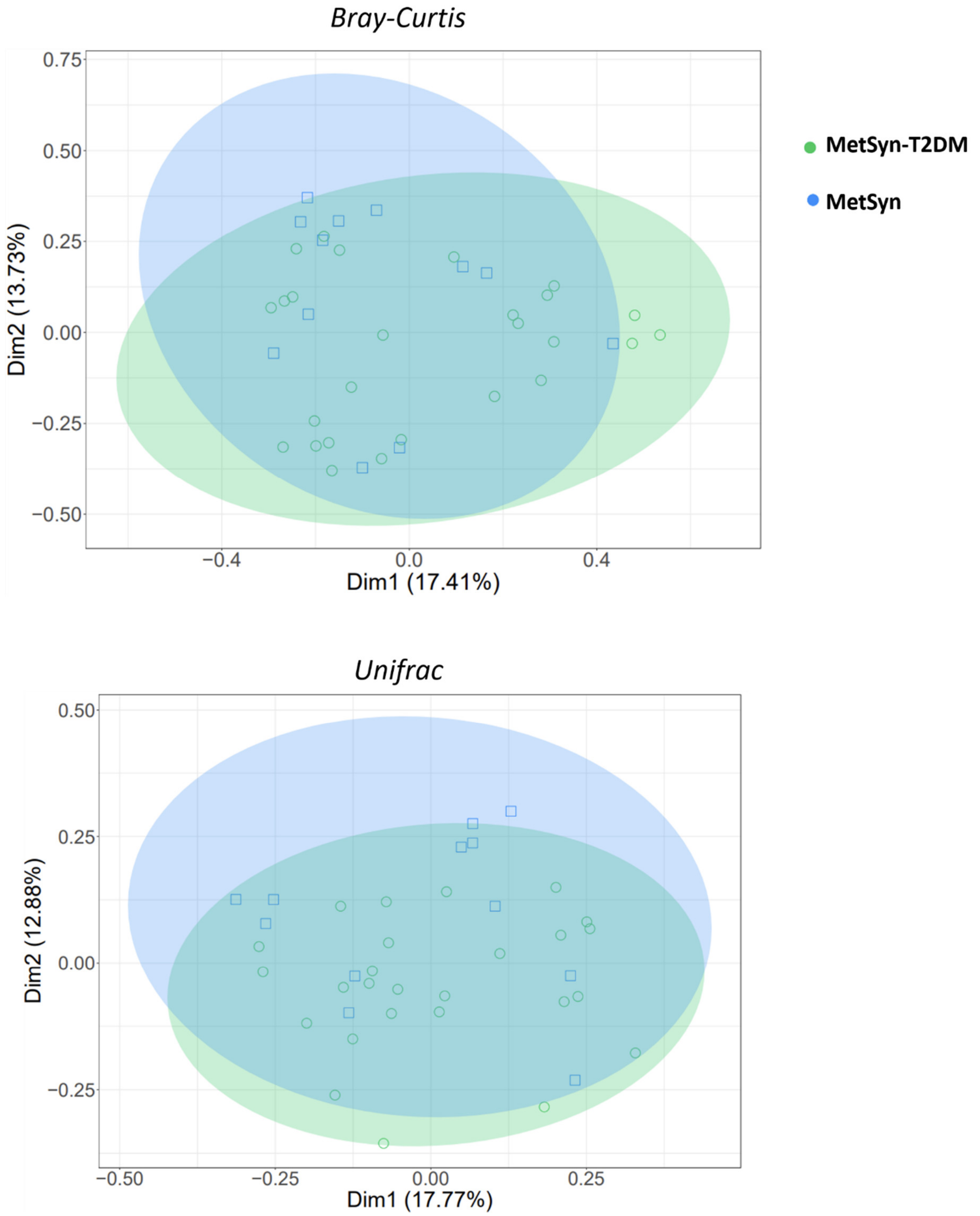

Figure S3. Beta diversity of the microbiome in MetSyn patients with and without diabetes measured by *Bray-Curtis* and *UniFrac* metrics

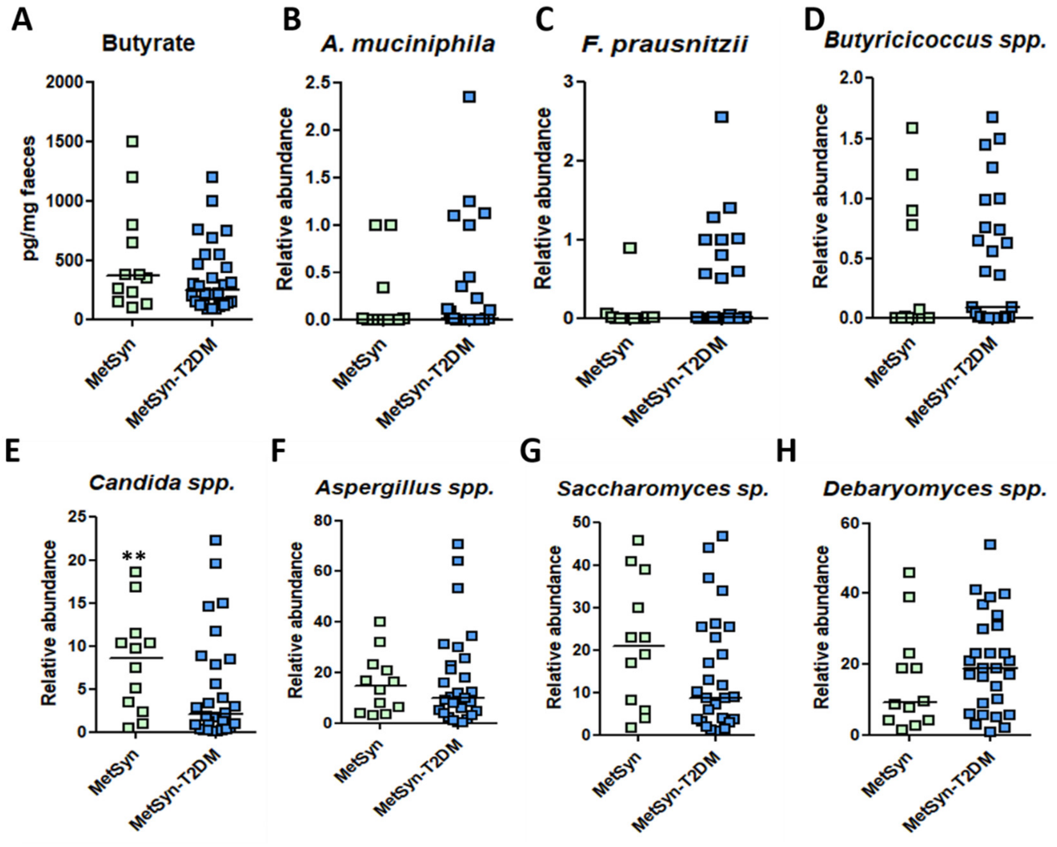

Figure S4. Microbiome changes in MetSyn patients with and without T2DM. (A) Butyrate quantification in fecal samples (p value= 0.3499); The relative abundance of *A. muciniphila (p value* = 0.2792) (**B**), *F. praunsitzii* (p value= 0.4816)(**C**), *Butyricicoccus* spp. (p value=0.3499) (**D**), *Candida spp. (**p value=0.0008)* (**E**), *Aspergillus spp. (p value*=0.5757) (**F**), *Saccharomyces spp.* (p value=0.4267) (**G**), and *Debaryomyces* spp. (p value=0.2815) (**H**) in fecal samples collected from MetSyn patients with and without T2DM

Table S4 – Subset analysis for statin treatment showing top subsets of OTUs along with the correlation of the beta diversity distances between these subsets and full OTU table. The last column shows PERMANOVA statistics for these subsets highlighting their discriminatory power. R^2 is the percentage variability of these subsets in terms of groups.

Group Comparison	Subset No	Subset	Correlation of Subset with Full Table (R)	PERMANOVA Subsets (Groups)
Statins, no statins	S1	*Clostridiales* + *Bacteroides* + *Bifidobacterium* + *Ruminococcaceae* + *Christensenellaceae* + *Lachnospiraceae* + *Ruminococcaceae UCG-002* + *Proteobacteria*	0.01815	$R^2 = 0.820$ ($p > 0.05$)
	S2	*Clostridiales* + *Bacteroides* + *Bifidobacterium* + *Ruminococcaceae* + *Christensenellaceae* + *Lachnospiraceae* + *Ruminococcaceae UCG-002*	0.01877	$R^2 = 0.780$ ($p > 0.05$)
	S3	*Clostridiales* + *Bacteroides* + *Bifidobacterium* + *Ruminococcaceae* + *Christensenellaceae* + *Lachnospiraceae* + *Ruminococcaceae UCG-002*	0.01898	$R^2 = 0.777$ ($p > 0.05$)

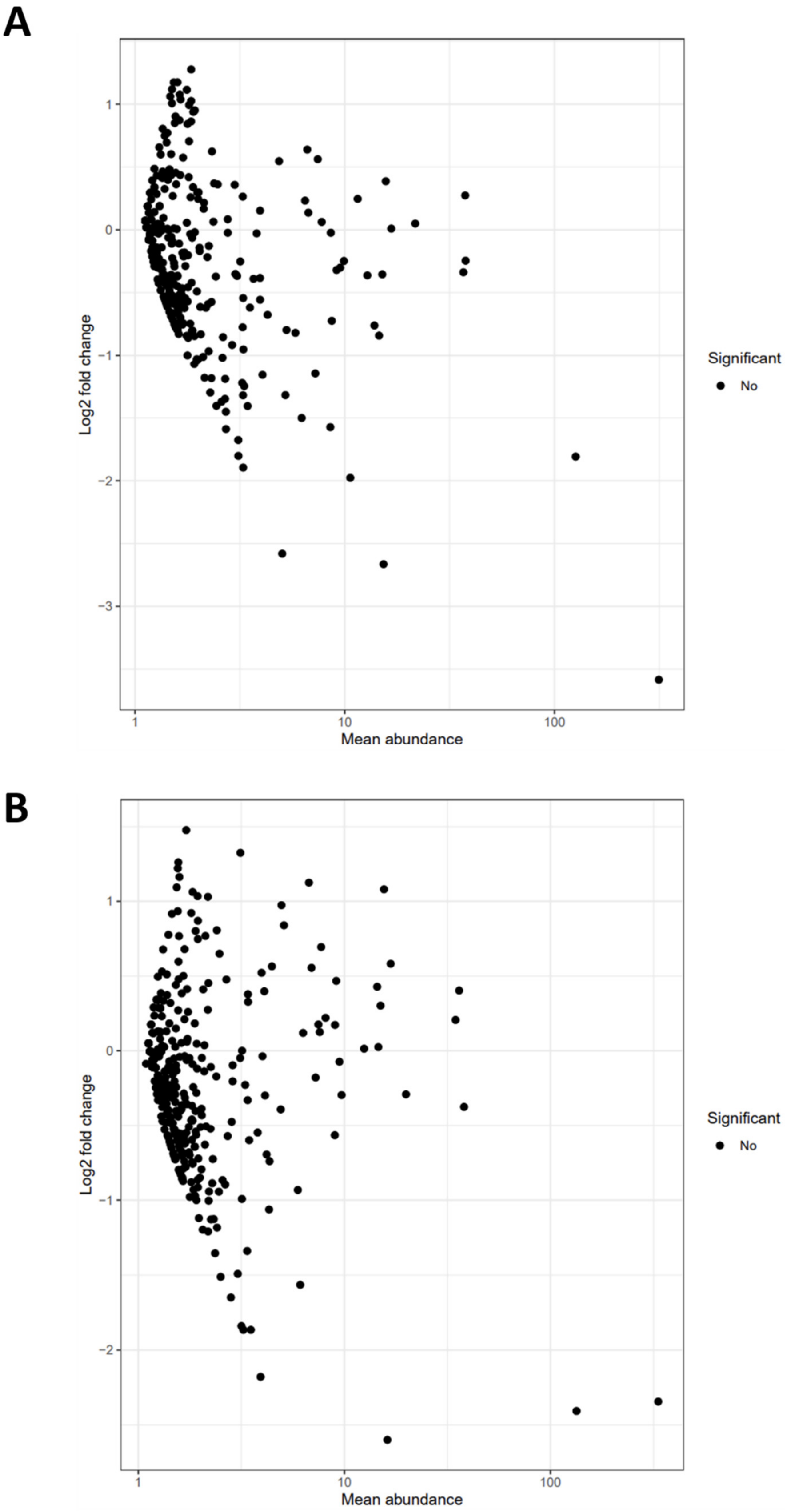

Figure S5. Taxa differential analysis in MetSyn patietns with T2DM (A) and MetSyn patients on statin treatment (B).

Table S5 – Subset analysis for metformin treatment showing top subsets of OTUs along with the correlation of the beta diversity distances between these subsets and full OTU table. The last column shows PERMANOVA statistics for these subsets highlighting their discriminatory power. R^2 is the percentage variability of these subsets in terms of groups.

Group Comparison	Subset No	Subset	Correlation of Subset with Full Table (R)	PERMANOVA Subsets (Groups)
Metformin, no metformin	S1	*Clostridiales* + *Bacteroides* + *Bifidobacterium* + *Ruminococcaceae* + *Ruminococcaceae UCG-002* + *Proteobacteria*	0.01815	R^2 = 0.835 ($p > 0.05$)
	S2	*Clostridiales* + *Bacteroides* + *Bifidobacterium* + *Ruminococcaceae* + *Ruminococcaceae UCG-002*	0.01877	R^2 = 0.804 ($p > 0.05$)
	S3	*Clostridiales* + *Bacteroides* + *Bifidobacterium* + *Ruminococcaceae* + *Ruminococcaceae UCG-002*	0.01898	R^2 = 0.768 ($p > 0.05$)

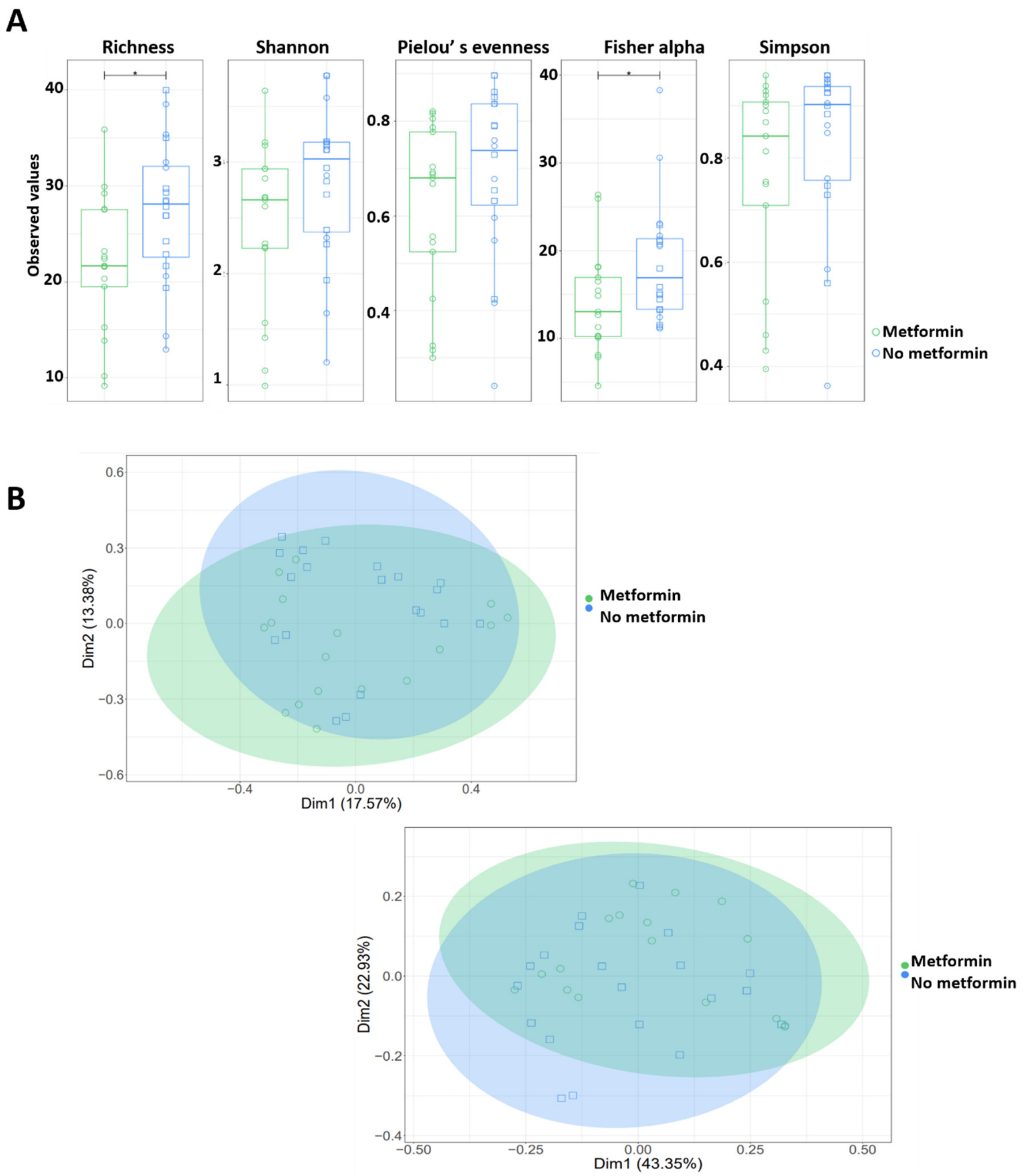

Figure S6. Microbial diversity and community structure for metformin vs. no metformin. A- Alpha diversity; B- beta diversity measured by *Bray-Curtis* (upper graph) and Weighted *UniFrac* (lower panel).
